# Adsorptive removal of losartan, bisphenol A, and triclosan in aqueous solutions using a graphene oxide-enhanced MOF-Zn composite

**DOI:** 10.1007/s11356-025-36961-9

**Published:** 2025-11-12

**Authors:** Ivon E. Valenzuela, Sebastián Valencia, Juan Carlos Muñoz-Acevedo, Ana Paula Silveira Paim, Elizabeth Pabón-Gelves

**Affiliations:** 1 Departamento de Química Fundamental, Universidade Federal de Pernambuco, Av. Prof. Moraes Rego, 1235––Cidade Universitária, Recife, PE 50670-901 Brazil; 2 Facultad de Ciencias, Departamento de Química, Universidad Nacional de Colombia Sede Medellín, Carrera 65 #59A-110, Medellín, Colombia; 3 Instituto de Química, Universidad de Antioquia, Calle 67 No. 53–108, Medellín, Colombia

**Keywords:** Metal–organic frameworks (MOFs), Graphene oxide, Removal performances, Adsorption capacity, Emerging pollutants, Water treatment

## Abstract

The increasing prevalence of emerging contaminants in aquatic systems represents a critical challenge to ecological and human health. Compounds such as losartan potassium (LO), bisphenol A (BPA), and triclosan (TN) have been identified as priority pollutants due to their environmental persistence, bioaccumulative behavior, and mechanistic involvement in endocrine disruption and the propagation of antimicrobial resistance. This study reports the synthesis of a zinc-based metal–organic framework functionalized with graphene oxide (MOF-Zn@GO) via a reflux method, using 2-aminoterephthalic acid and graphene oxide, designed for the adsorption of LO, BPA, and TN. The MOF-Zn@GO composite was characterized by XRD, FTIR, Raman, XPS, TGA, BET, and zeta potential analysis. XRD patterns, along with TGA results, confirmed that the crystalline structure remained thermally stable after GO incorporation. FTIR and Raman spectra revealed functional groups (–OH, –COOH) and π-conjugated domains from GO. In addition, XPS analysis showed an increase in the signals associated with C–O and C = O bonds, supporting the incorporation of GO into the MOF–Zn structure. The material exhibited a specific surface area of 329.7 m^2^ g⁻^1^ and an average pore size of 2.1 nm. The point of zero charge (pHpzc) was determined to be 6.8, indicating favorable surface properties for contaminant adsorption. Adsorption experiments revealed maximum capacities (*Qe*) of 395 mg g⁻^1^ for LO, 275 mg g⁻^1^ for BPA, and 300 mg g⁻^1^ for TN, attributed to the synergistic effects of hydrogen bonds and π–π interactions provided by graphene oxide. Quantification of pollutant removal was achieved using high-performance liquid chromatography (HPLC) with UV–Vis detection, confirming the high efficiency and analytical precision of the process. Kinetic studies showed that the adsorption process followed pseudo-second-order kinetics and was best described by the Freundlich isotherm model, indicating multilayer adsorption on a heterogeneous surface. Optimal removal was achieved at pH 4.5 for LO and pH 7.5 for BPA and TN, with an adsorbent dosage of 10 mg, the initial pollutant concentration of 15 mg L⁻^1^, and an eluent volume of 600 μL. The excellent performance of the adsorbent was demonstrated by the high removal percentages of LO 99.3%, BPA 90.1%, and TN 97.2%. The MOF-Zn@GO composite maintained over 85% of its initial adsorption efficiency after five regeneration cycles, with negligible loss of active sites and excellent structural stability. These findings underscore the material’s potential as a robust and reusable adsorbent for the efficient removal of emerging contaminants from aquatic systems, thus contributing to cleaner water management strategies.

## Introduction

The escalating presence of emerging pollutants (EPs) in aquatic systems is raising serious concerns due to their persistence, potential for bioaccumulation, and wide-ranging toxic effects, which collectively threaten ecosystem integrity and human health. EPs comprise a broad spectrum of compounds, including pharmaceuticals, plasticizers, and antimicrobial agents (Du et al. 2022; Li et al. 2023). These contaminants are primarily introduced through industrial effluents, municipal wastewater, and agricultural runoff and are associated with adverse biological effects (Fachina et al. 2023; Hu et al. 2024).

Among them, losartan potassium (LO), bisphenol A (BPA), and triclosan (TN) are frequently detected emerging contaminants in surface water, groundwater, and even drinking water sources (Hassan et al. 2023). These compounds represent distinct classes of pollutants with diverse physicochemical properties, including differences in molecular size, polarity, and functional groups. LO is a polar pharmaceutical compound with low biodegradability. As a widely prescribed antihypertensive, it is excreted mostly unmetabolized and has been reported at concentrations ranging from 0.17 to 36.5 ng/L in surface waters, 5 ng/L in drinking water, 1–2 µg/L in treated effluents, and up to 4.5 µg/L in raw hospital wastewater (Reque et al. 2021). These levels raise concern due to their persistence and potential to induce oxidative stress and endocrine disruption in aquatic organisms (Medici et al. 2024). BPA is a high-production-volume industrial monomer used in the manufacturing of polycarbonate plastics, epoxy resins, and thermal paper. It has been recorded in wastewater effluents at 16–1465 ng/L, and in surface waters at 170–3113 ng/L. In wastewater treatment plants (WWTPs), influent concentrations range from 5.2 to 138 ng/L, and concentrations in sludge feeds reach up to 1960 ng/L (Caban and Stepnowski 2020). BPA is a well-known endocrine-disrupting compound (EDC), capable of interfering with reproductive and developmental processes even at sub-ng/L levels (Geens et al. 2015). TN is a chlorinated antimicrobial agent commonly found in personal care and household products. It is frequently released into aquatic environments due to high consumption and improper disposal. TN is highly persistent and can be transformed into chlorinated dioxins, which are significantly more toxic. Its concentrations in effluents from municipal wastewater treatment plants range from 443 to 1757 ng/L (Mohan and Balakrishnan 2019), and it has been linked to antimicrobial resistance and toxicity to algae, invertebrates, and fish (Lee et al., 2024; Dar et al. 2022).

Conventional water treatment methods, including activated sludge, coagulation, flocculation, and chlorination, are often insufficient for removing these pollutants due to their low molecular weight, hydrophilicity, and chemical stability (Mishra et al. 2019; Santhappan et al. 2024; Ogwu et al. 2024). As a result, advanced treatment processes, especially adsorption-based technologies, are increasingly being explored (Liu et al. 2024a, b). Traditional adsorbents such as activated carbon, zeolites, and biopolymers have shown potential but are often limited by low selectivity, mechanical instability, limited regeneration capacity, and reduced efficiency at trace concentrations (Hussain et al. 2025; Sharma et al. 2024; Khan et al. 2025; Lladó et al. 2025).

Metal–organic frameworks (MOFs) have emerged as highly promising porous materials for pollutant adsorption owing to their exceptional surface area, tunable pore structures, and chemical versatility (Marghade et al. 2024; Kaur et al. 2023). MOFs are crystalline networks composed of metal ions or clusters coordinated with organic ligands, allowing diverse host–guest interactions including π–π stacking, hydrogen bonding, electrostatic forces, and acid–base interactions (Rana et al. 2024; Sundararaman et al. 2024). In particular, zinc-based MOFs such as ZIF-8, MOF-5, and MOF-74 have demonstrated high stability and affinity for various pollutants, including pharmaceuticals and plasticizers (Lu et al. 2023). However, their practical application is often limited by issues such as aggregation, poor dispersibility in water, and limited mechanical integrity (Ploychompoo et al. 2021).

Recent studies have focused on integrating MOFs with graphene oxide (GO), forming MOF@GO composites that combine the porosity and selectivity of MOFs with the high surface area, hydrophilicity, and functional group diversity of GO stacking (Dreyer et al. 2010). GO’s oxygenated functionalities (e.g., –OH, –COOH, epoxy) facilitate strong interactions with a wide range of contaminants and enhance dispersion in aqueous media (Zhao et al. 2011). Buitrago Sánchez et al. (2024) synthesized GO from coal and coke for ciprofloxacin adsorption. Moreover, GO improves mechanical robustness and prevents MOF agglomeration, enhancing adsorption kinetics and overall performance (Zhou et al. 2021). Several studies have demonstrated that the incorporation of GO into MOFs significantly improves adsorption capacities for dyes and pharmaceutical residues (Mkilima et al. 2024). Anum et al. (2023) demonstrated high adsorption efficiency of GO/MOF-5 composites for moxifloxacin (up to 95%), attributed to synergistic interactions. The synergistic combination of MOFs and GO enhances adsorption interactions, significantly improving the composite’s capacity to capture a wide range of contaminants. This highlights their potential as advanced materials for water purification. (Qi et al. 2023; Shahabi et al. 2024).

In this study, a zinc-based metal–organic framework functionalized with graphene oxide (MOF-Zn@GO) was synthesized to evaluate its adsorption capacity and removal of three EPs: LO, BPA, and TN. These pollutants were selected due to their prevalence in water sources and their known adverse effects on both environmental and human health. The adsorption performance of MOF-Zn@GO in aqueous solutions was evaluated, along with its reusability and stability, to explore its potential as a sustainable and high-performance material for water remediation. The findings of this study contribute to the ongoing development of advanced multifunctional adsorbents and provide valuable insights into the design of efficient water treatment technologies aimed at mitigating water pollution challenges.

## Experimental section

### Materials

Zinc nitrate hexahydrate (Zn(NO_3_)_2_·6H_2_O, ≥ 99%, analytical grade), losartan potassium (LO, C_22_H_22_ClKN_6_O, ≥ 98%, pharmaceutical grade), bisphenol A (BPA, C_15_H_16_O_2_, ≥ 99%, reagent grade), and triclosan (TN, C_12_H_7_Cl_3_O_2_, ≥ 97%, analytical standard) were purchased from Sigma-Aldrich; 2-aminoterephthalic acid (C₈H₅NO₄, 99%, reagent grade) and graphite powder (< 20 µm, synthetic, ≥ 99.5%) were obtained from Alfa Aesar. *N*,*N*-dimethylformamide (DMF, (CH_3_)_2_NCHO, ≥ 99.8%, HPLC grade) and acetonitrile (ACN, CH₃CN, ≥ 99.9%, HPLC grade) were supplied by Scharlau (Spain). All aqueous solutions were prepared using Milli-Q ultrapure water (Direct-8 purification system, resistivity > 18 MΩ cm, Millipore). Other reagents, such as hydrochloric acid (Merck), sulphuric acid (Merck), and sodium hydroxide (Merck), were analytical grade.

### Synthesis of MOF-Zn@GO

For the synthesis of MOF-Zn@GO, 3.05 g of 2-aminoterephthalic acid was dissolved in 40 mL of DMF, and the solution was placed in a 250-mL round-bottom flask equipped with magnetic stirring. Separately, 0.070 g of GO was dissolved in 20 mL of DMF and added to the initial solution. The GO was synthesized using a modified Hummers method, as described by Akhavan et al. (2014). Graphitized powder (1.0 g) was dispersed in 50 mL concentrated H₂SO₄ and heated at 80 °C for 24 h. After cooling in an ice bath, 1.0 g NaNO_3_ was added and stirred for 1 h; 6.0 g KMnO_4_ was slowly added under vigorous stirring and reacted for 4 h, followed by stirring at 35 °C for 1 h and cooling to room temperature. The suspension was diluted with 100 mL of distilled water, and excess permanganate was reduced with H_2_O_2_. The product was purified by centrifugation to remove residual acids and impurities. The mixture of 2-aminoterephthalic acid and GO was then subjected to reflux at 90 °C for 4 h. Subsequently, 1.0 g of Zn(NO_3_)_2_·6H_2_O dissolved in 40 mL of DMF was added to the solution. The reaction mixture was heated under reflux for 4 h. Upon completion, the resulting product was collected by vacuum filtration, washed three times with DMF (10 mL each), and three times with water (10 mL each), and then dried at 80 °C for 4 h. For comparison, MOF-Zn was synthesized under identical conditions to those used for MOF-Zn@GO, excluding the addition of GO.

### Characterization

#### Structural, thermal, and morphological characterization

XRD patterns of the MOF-Zn@GO solid samples in powder form were collected using a Malvern-PANalytical diffractometer with CuKα radiation at room temperature, covering an angular range of 2θ = 5–90°. FTIR spectra were obtained using a Bruker FTIR model IFS 66 spectrometer over a wavenumber interval of 4000 to 500 cm⁻^1^, applying the KBr pressed-disk technique. XPS measurements were conducted using a SPECS-PHOIBOS 150 1D-DLD system, with monochromatic Al-Kα X-ray (1486.7 eV, 13 kV, 100 W). The graphene oxide (GO), MOF-Zn, and MOF-Zn@GO were evaluated using a PerkinElmer Raman spectrometer over the wavelength range of 2000–100 cm⁻^1^.

TGA was performed under a nitrogen atmosphere using a Shimadzu TGA-60 system, within a temperature range of 25–800 °C at a heating rate of 10 °C min⁻^1^ for the bulk sample and 5 °C min⁻^1^ for smaller samples. The morphology of MOF-Zn and MOF-Zn@GO was examined using a JEOL JSM 5910LV SEM operating at 15 kV and equipped with a Bruker EDX system for elemental analysis. Samples were stored in a desiccator for one day and fixed onto aluminum supports, followed by metallization with a gold layer (10–20-nm thickness) to reduce charging effects.

#### Surface and textural properties

Surface area measurements were conducted using nitrogen adsorption–desorption isotherms following the method of Rouquerol et al. 2007 using an ASAP 2020 PLUS-3030 system. The samples were pretreated at 180 °C for 240 min under high vacuum conditions to degas. The adsorption/desorption isotherms were collected within a relative pressure range of 0.1–0.998 *P*/*P*₀, with at least 50 data points.

The point of zero charge (pHpzc) of MOF-Zn@GO was determined using the pH-drift method. A series of 0.01 M NaCl solutions (50 mL) was adjusted to initial pH values ranging from 1 to 12 by adding 0.1 M HCl or 0.1 M NaOH. Then, 50 mg of the material was added to each solution, and the suspensions were stirred for 24 h at room temperature. After equilibration, the initial and final pH values of the solutions before and after the adsorption process were measured and plotted.

Zeta potential measurements of MOF-Zn@GO were performed using a Zetasizer 2680-ZS90 size analyzer (Malvern Panalytical Ltd) at different pH values (3, 4.5, 6, 7.5, and 9). The pH was adjusted by dropwise addition of 0.1 M HCl or 0.1 M NaOH under constant stirring until the desired value was reached. The pH of each solution was then confirmed, and suspensions were allowed to stabilize for 10 min before measurement. Solutions with 1% w/v concentrations were sonicated for 5 min before measurement. Each sample was analyzed in triplicate at 25 °C. The refractive index used for the measurements was that of silica, and the detection angle was 90°.

### Adsorption experiment

For the adsorption experiments, 10 mg of the sorbent material (MOF-Zn@GO) was conditioned with 1 mL of acetonitrile (ACN) and water to activate the adsorbent surface and remove residual impurities or molecules trapped within the pores of the material. Subsequently, 10 mL of a standard or sample solution containing all emerging pollutants (EPs) at a concentration of 15 mg L⁻^1^ was loaded onto the sorbent. The physicochemical properties of EPs are summarized in Table 1. Individual standard solutions were prepared at 200 mg L⁻^1^ in ACN and diluted with water to achieve the daily working concentrations.

**Table 1:**
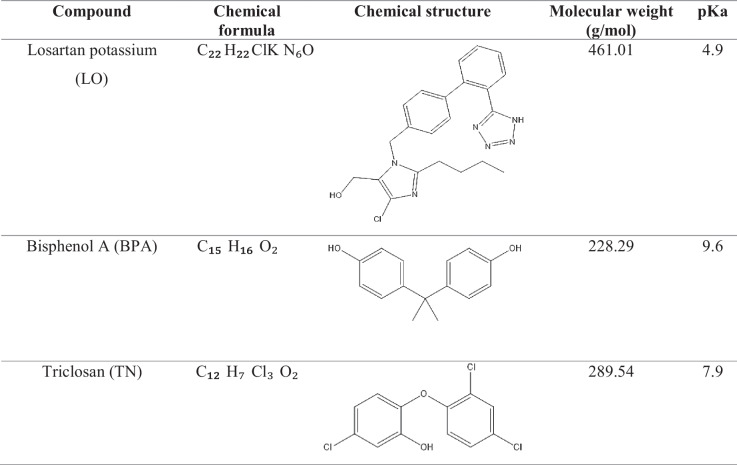
Physicochemical properties of EPs

**Fig. 1:**
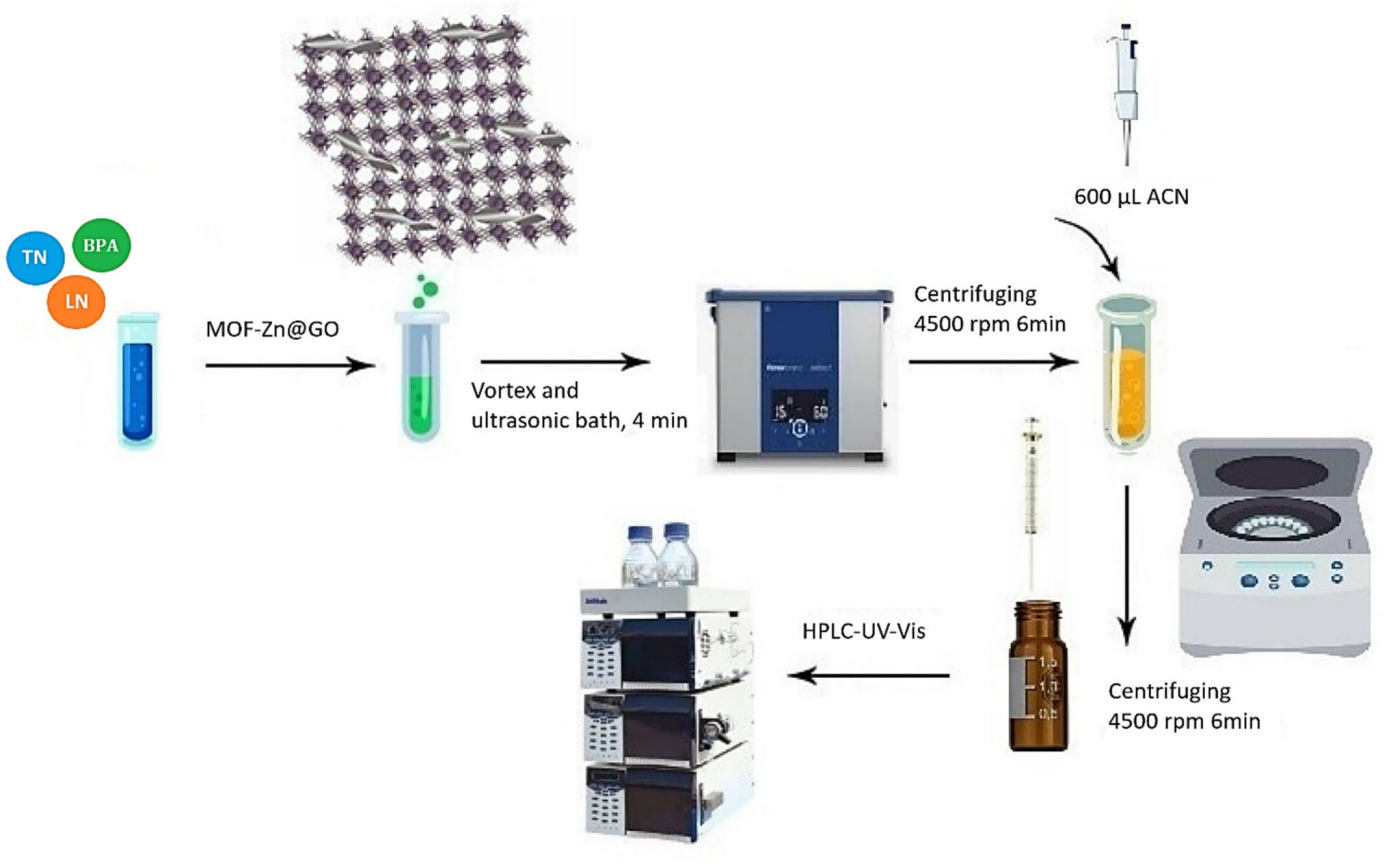
Adsorption scheme of LO, BPA, and TN onto MOF-Zn@GO

The extraction process was assisted by ultrasound for 4 min, followed by 1 min of vortex agitation and centrifugation at 4500 rpm for 6 min. After centrifugation, the supernatant was carefully removed from the aqueous phase, and the retained EPs were desorbed using 200 μL of ACN, applied in three successive steps. Extraction devices were regenerated by rinsing thoroughly with water to remove excess ACN. The sorbent can be reused by applying the same procedure. Before injection into the high-performance liquid chromatography (HPLC) with a UV–Vis detector, desorbed fractions were filtered through a 0.22-μm polytetrafluoroethylene (PTFE) membrane. The simultaneous adsorption capacity of the prepared porous material for micropollutants was evaluated using 10 mL of a composite solution at concentrations ranging from 50 to 250 mg L⁻^1^, with an extraction time of 24 h. The concentration of contaminants remaining after extraction was measured using HPLC–UV–Vis. All extraction experiments were performed in triplicate. Figure 1 shows a simplified scheme of the procedure.

The maximum adsorption capacity (*Q*_*e*_) was calculated using Eq. 1:

1 $${Q}_{e}=\frac{({C}_{0}-{C}_{e})}{m }\times V$$

where *m* is the amount of sorbent (mg), *C₀* and *Cₑ* are the initial and equilibrium concentrations of analytes (mg L⁻^1^), and *V* is the volume of the solution (L).

The removal efficiency was calculated according to the following Eq. 2:

2 $$\% \text{Removal}=\frac{({C}_{0}-{C}_{e})}{{C}_{0} } \times 100$$

The adsorption parameters for the removal of LO, BPA, and TN using MOF-Zn@GO were also evaluated using univariate analysis. Several factors, including pH, initial pollutant concentration, sorbent mass, and eluent volume, were systematically evaluated to maximize adsorption efficiency. The effect of pH on adsorption was investigated by adjusting the solution pH between 3.0, 4.5, 6.0, 7.5, and 9.0. This parameter is critical as it influences the surface charge of MOF-Zn@GO and the ionization state of the pollutants, thereby affecting adsorption capacity. The initial concentration of EPs was varied between 5 and 25 mg L⁻^1^ to assess the impact of pollutant load on the adsorption performance. The mass of the sorbent was studied by varying the amount of MOF-Zn@GO used in the adsorption process from 5 to 20 mg. Finally, the volume of the eluent (ACN) required for efficient desorption of the pollutants was tested between 200, 400, 600, 800, and 1000 µL.

### HPLC conditions

The separation of the analytes was performed using a 1200 HPLC system (Agilent Technologies) equipped with a UV–Vis detector. The separation was achieved on an Eclipse XDB C18 column (150 × 4.6 mm, 5-μm particle size) under gradient elution mode. The mobile phase consisted of two solvent systems: (A) water-ACN (40:60) and (B) phosphate buffer (pH 6.6), ACN (65:35) with flow rates of 0.8 mL min⁻^1^ and 1.0 mL min⁻^1^, respectively. The detection wavelength was set to 265 nm for LO, while BPA and TN were detected at 220 nm to improve sensitivity. This method provided effective separation and quantification of the target emerging pollutants.

### Performance evaluation

To evaluate the adsorption efficiency of MOF-Zn@GO for the removal of emerging pollutants (EPs) such as LO, BPA, and TN, a series of solutions with concentrations ranging from 0.1 to 50 µg mL⁻^1^ was prepared to determine the working range for high-performance liquid chromatography coupled with UV–Visible detection (HPLC–UV–Vis). The limits of detection (LOD) and limits of quantification (LOQ) were established for solutions spiked with low concentrations of the target EPs, following the protocol described by Singh et al. (2015). Method accuracy was evaluated by calculating the relative standard deviation (RSD) for EP concentrations of 1 µg mL⁻^1^ across five replicates (*n* = 5) under optimized experimental conditions. The coefficient of variation must be ≤ 2% for all concentrations.

The recovery rates of the EPs were calculated by dividing the amount extracted from the spiked samples by the initial standard concentration, with statistical significance set at 5%. Additionally, the adsorption performance of MOF-Zn@GO was tested in different water matrices, including ultrapure and tap water, using the standard addition method. The samples were spiked with EPs to reach a final concentration of 15 µg mL⁻^1^, and the adsorption efficiency of the material was investigated under optimized experimental conditions.

### Recyclability test

The recyclability and reusability of MOF-Zn@GO were evaluated by testing its performance in the adsorption of emerging pollutants (EPs) over five consecutive cycles under optimized conditions. Following each extraction cycle, the sorbent was thoroughly regenerated by washing it three times with 1 mL of ACN and water (H₂O) to effectively remove any EPs adsorbed onto the surface. This regeneration process ensured that the MOF-Zn@GO could be reused in subsequent adsorption and desorption cycles without significant loss of performance.

## Results and discussion

### Characteristic properties of MOF-Zn@GO composites

#### Structural, thermal, and morphological characterization

A zinc-based metal–organic framework (MOF-Zn) functionalized with graphene oxide (GO), denoted as MOF-Zn@GO, was synthesized using a reflux method, with 2-aminoterephthalic acid serving as the organic ligand.

**Fig. 2:**
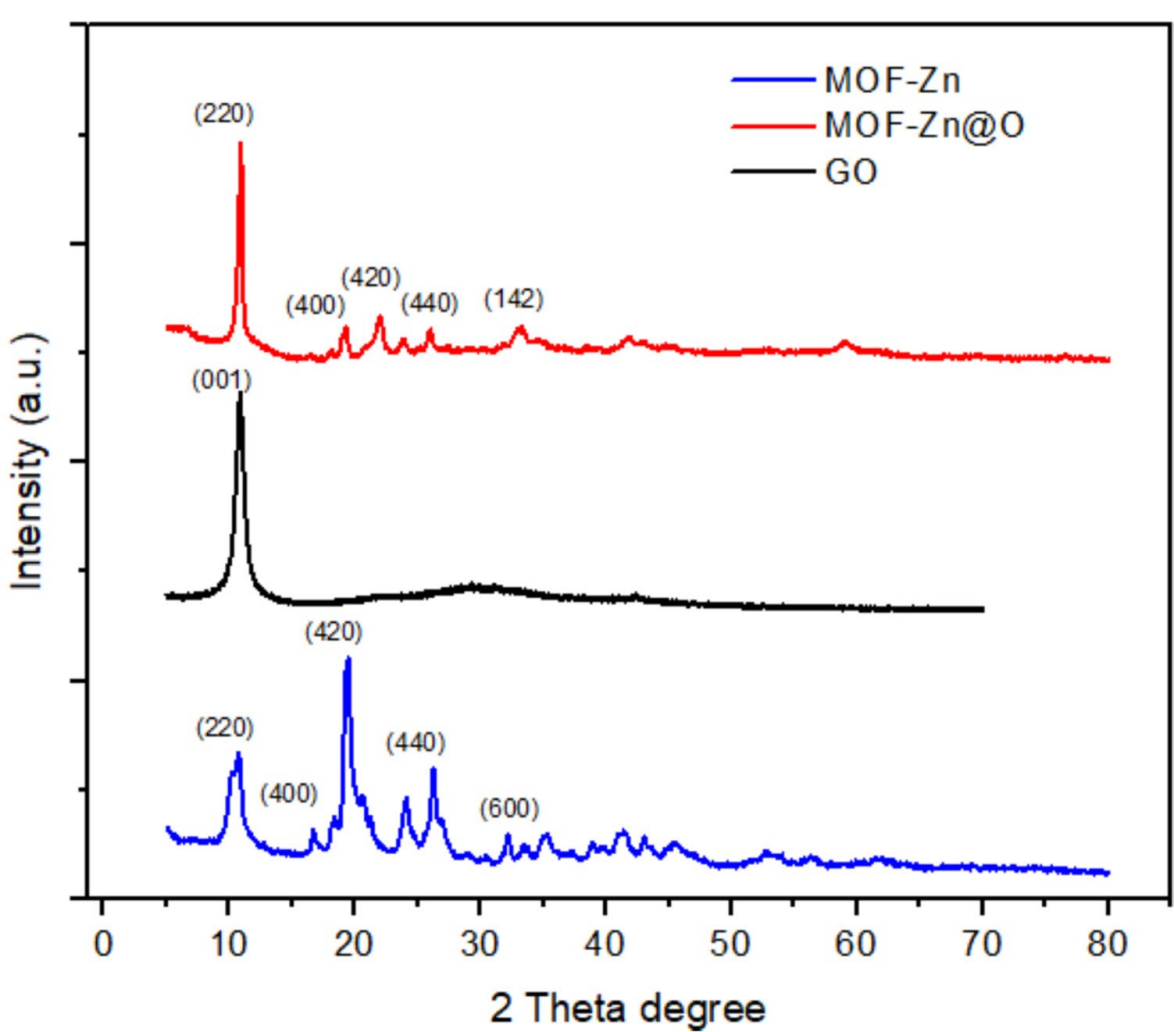
XRD patterns of the MOF-Zn, MOF-Zn@GO, and GO samples

The structural attributes of GO, MOF-Zn, and MOF-Zn@GO composite were characterized through X-ray diffraction (XRD), as shown in Fig. 2, which reveals the crystalline nature and phase purity of the synthesized material. The incorporation of GO within the MOF structure was confirmed by distinct diffraction patterns, where characteristic peaks from both the MOF-Zn and MOF-Zn@GO frameworks are evident. The XRD pattern of GO exhibits a distinct peak at 2θ = 11°, assigned to the (001) plane. This peak reflects the increased interlayer spacing due to the introduction of oxygen-containing functional groups during oxidation. The intensity of this peak correlates with the degree of oxidation in the GO structure. The XRD pattern of MOF-Zn@GO exhibits multiple sharp peaks with high intensity, indicating a well-defined crystalline structure. Peaks at 2θ = 10.6°, 19.4°, 24.0°, 26.2°, and 32.1° correspond to the (220), (400), (420), (440), and (142) planes, respectively, which are consistent with the MOF-Zn structure (Liu et al. 2024a, b). The MOF-Zn@GO composite also displays a new broad peak around 2θ = 11°, confirming the presence of GO within the hybrid material. The degree of crystallinity (%*Xc*) of the synthesized materials was calculated using the following Eq. 3:

3 $$\%Xc= \frac{\text{Area under crystalline peaks}}{\text{Total area }(\text{crystalline }+\text{ amorphous})} \times 100$$

The areas were obtained from the XRD patterns by integrating the intensities of the crystalline peaks and the amorphous background. According to this analysis, the MOF-Zn sample exhibited a crystallinity of 75%, while the MOF-Zn@GO composite showed a slightly lower crystallinity of 66%. This decrease can be attributed to the incorporation of GO, which disrupts the long-range order of the metal–organic framework, leading to a partial reduction in crystallinity. These findings are in agreement with previous reports (Gwardiak et al. 2019; Patel and Parsania 2018).

**Fig. 3:**
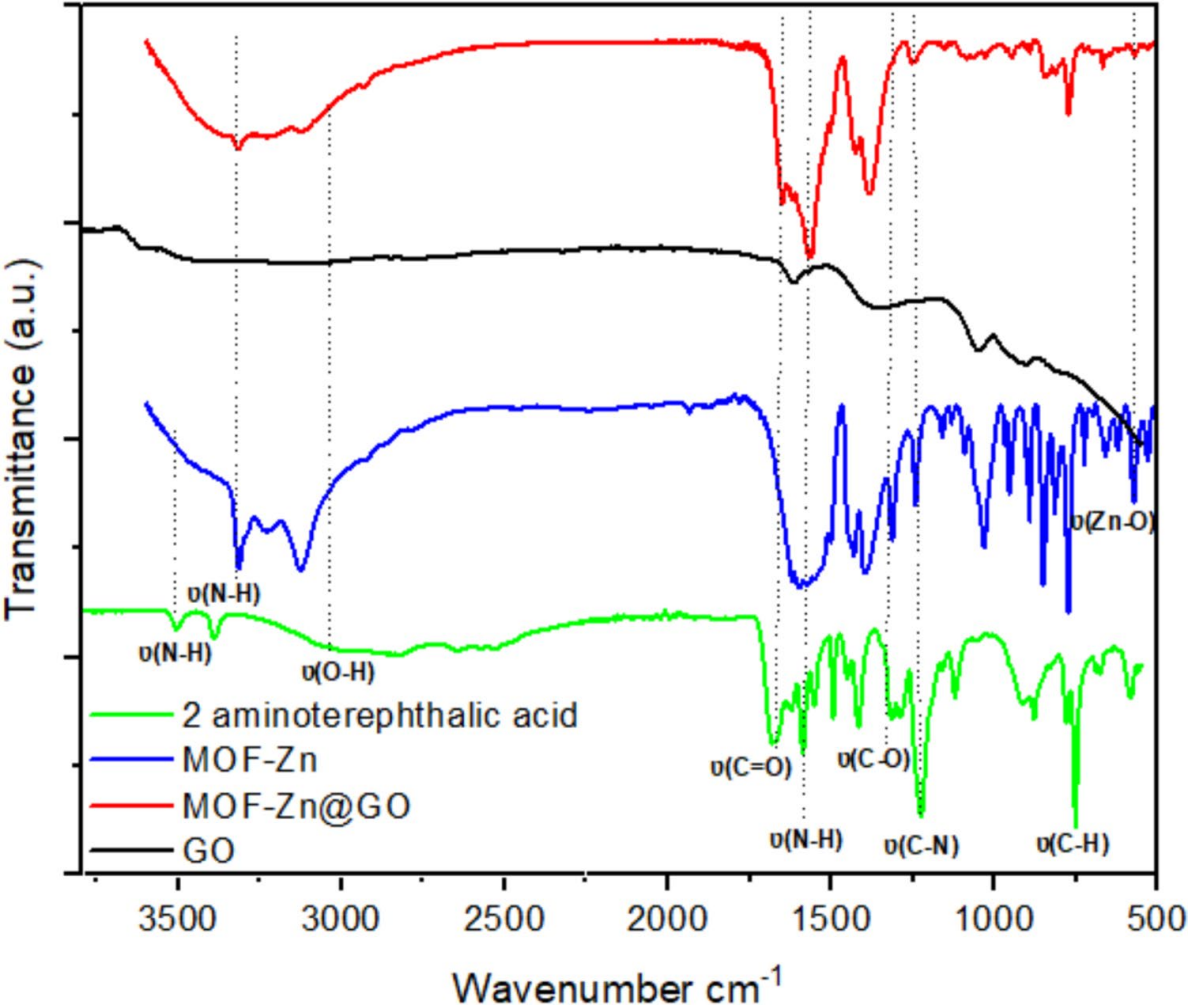
FTIR spectra of 2-aminoterephthalic acid, MOF-Zn, GO, and MOF-Zn@GO

Figure 3 shows FTIR spectra of 2-aminoterephthalic acid, MOF-Zn, GO, and MOF-Zn@GO, where the interactions between the MOF framework and GO are further elucidated. The spectrum of 2-aminoterephthalic acid displays characteristic peaks at ~ 3470 and ~ 3380 cm⁻^1^ corresponding to the asymmetric and symmetric stretching vibrations of N–H groups, as well as a band near 1670 cm⁻^1^ associated with C = O stretching of the carboxylic acid group, while bands in the range of ~ 1222 cm⁻^1^ are attributed to C–N (Zhang et al. 2022). The GO spectrum shows broad absorption around 3400 cm⁻^1^ due to O–H stretching vibrations from hydroxyl groups, and a band near 1648 cm⁻^1^ corresponding to C = O stretching of carboxylic acid and carbonyl functionalities (Abbasi et al. 2023).

The MOF-Zn spectrum exhibits characteristic bands of the metal–organic framework structure. The disappearance of free carboxylic acid peaks and the emergence of symmetric and asymmetric COO⁻ stretching at ~ 1600 and asymmetric C–O ~ 1380 cm⁻^1^ confirm the coordination of carboxylate groups to Zn^2^⁺ ions (Arya et al. 2023). Additionally, the N–H stretching vibrations remain visible, indicating partial retention of amine functionalities. Bands in the fingerprint region (1300–700 cm⁻^1^) correspond to C–N, Zn–O, and aromatic C–H bending modes (Liu et al. 2024a, b). In the MOF-Zn@GO composite, most of the characteristic MOF-Zn peaks are retained, suggesting that the framework structure is preserved after functionalization. However, notable shifts in the carboxylate and amine region (e.g., bands at ~ 1600 and ~ 1380 cm⁻^1^) indicate possible interactions between the MOF and GO, such as hydrogen bonding or π–π stacking. The broadening of the O–H stretching band and reduction of the C = O signal from GO suggest successful integration of MOF-Zn onto the GO surface, confirming that the GO functionalization enhances the MOF’s properties while preserving its stability (Valenzuela et al. 2024). Additionally, the incorporation of GO into the MOF framework introduces new functional groups that potentially enhance the adsorption affinity of the material for emerging contaminants (EPs) due to interactions such as hydrogen bonds and π–π interactions between the functional groups of GO and the target pollutants (Chen et al. 2024).

**Fig. 4:**
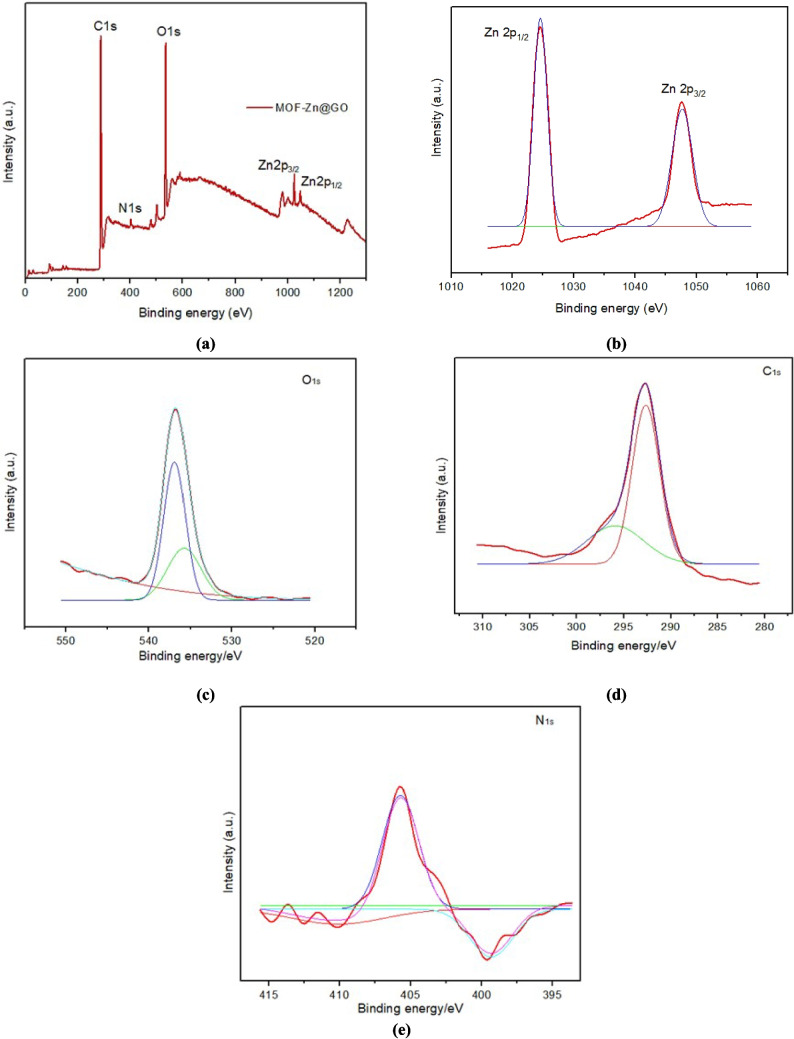
**a** XPS of MOF-Zn@GO, **b** Zn spectrum, **c** O spectrum, **d** C spectrum, and **e** N spectrum

The XPS spectrum of MOF-Zn@GO, depicted in Fig. 4a, confirms the presence of C, N, O, and Zn elements within the composite. The high-resolution XPS spectrum of Zn 2p, shown in Fig. 4b, provides insight into the oxidation state and bond environment of zinc in the MOF framework. The Zn 2p level is split into two distinct peaks: 2p₁/₂ at 1047.8 eV and 2p₃/₂ at 1024.6 eV, corresponding to the spin–orbit split components of Zn 2p. The observed energy difference of 23.2 eV between the 2p₁/₂ and 2p₃/₂ peaks is consistent with the typical Zn^2^⁺ oxidation state, confirming the expected coordination environment of zinc within the composite. The XPS O1s spectra have two peaks at 536.6 and 539.6 eV (Fig. 4c), corresponding to the C–O and Zn–O/C = O bonds, respectively. The C1s peaks at 292.6 and 289.6 eV corresponds to carboxyl and phenyl groups, further verifying the incorporation of GO functional groups within the MOF structure (Fig. 4d). Additionally, in the Fig. 4e, the N1s peak peaks at 405.6 and 406.6 eV are characteristic of the N–C bond, suggesting the coordination of nitrogen from the solvent within the MOF-Zn@GO matrix (Bi et al. 2022 and Wei et al. 2022). This spectral information highlights the effective integration of GO with Zn within the MOF, which is critical for the material’s structural integrity and functional properties.

**Fig. 5:**
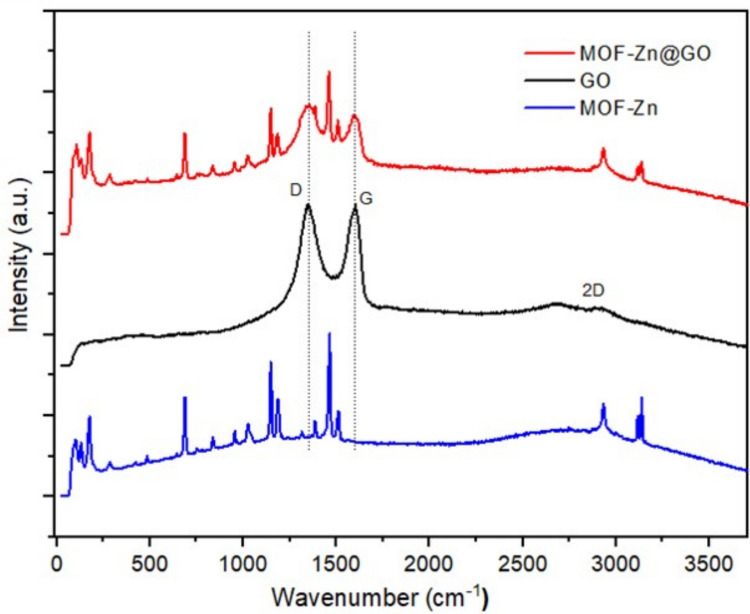
Raman spectra of MOF-Zn, MOF-Zn@GO, and GO

The functionalization of MOF-Zn with GO was investigated through Raman spectroscopy, as illustrated in Fig. 5. The Raman spectrum of GO reveals two prominent bands: the G band at 1710 cm⁻^1^, attributed to the *sp*^2^ hybridized carbon domains, and the D band at 1442 cm⁻^1^, which corresponds to structural defects and edge distortions within the carbon framework. The intensity ratio of the D to G bands (I_D_/I_G_) serves as a metric for evaluating the degree of disorder and structural integrity within GO. In this study, an *I*_*D*_/*I*_*G*_ ratio of ≈ 1.0 suggests that the reduction process with KMnO_4_ induces notable alterations in the GO structure, while preserving essential functional groups necessary for subsequent interactions within the MOF matrix (Gordi et al. 2020).

In the MOF-Zn@GO composite, the G band at 1601 cm^−1^ and the D band at 1340 cm^−1^ are observed with a marked decrease in intensity. This reduction in intensity points to a significant modification of the *sp*^2^ carbon domains, reflecting a decrease in the defect density and partial restoration of the graphitic network upon integration with the MOF structure. The diminished intensity and shifted positions of these bands suggest substantial structural and electronic modifications in the GO, as it is incorporated into the MOF-Zn matrix. This integration likely enhances the composite’s stability and facilitates electronic interactions within the framework, potentially enhancing its adsorption and catalytic properties (Shah et al. 2021).

The average lateral crystallite size (La) was calculated to be approximately 19.2 nm by applying Eq. 4 with a laser wavelength of 532 nm.

4 $$La(\text{nm})=\frac{2.4\times {10}^{-10})({\lambda }^{4})}{\frac{{I}_{D}}{{I}_{G}}}$$

In this equation, *La* represents the average lateral crystallite size in nanometers (nm), *λ* is the laser wavelength used in the Raman spectroscopy (in nm), and *I*_*D*_/*I*_*G*_ corresponds to the intensity ratio of the D and G bands, respectively. The resulting value indicates the presence of relatively well-ordered graphitic domains within the GO structure.

The intensity ratio *I*_2*D*_/*I*_*G*_ was estimated to be approximately 0.6, which is indicative of few-layer GO, typically corresponding to 3–5 layers. The moderate intensity and broad shape of the 2D band further support the presence of multilayer GO rather than monolayer or bilayer structures. These spectral features may also suggest partial reduction, improved structural ordering, or a decreased number of layers. These characteristics are indicative of increased π–π conjugation and a decreased presence of oxygen-containing functional groups or defects (Akhavan et al. 2014). Raman analysis indicates decreased defect density and partial graphitic network restoration upon GO incorporation into MOF-Zn. This structural ordering enhances π–π stacking interactions and modifies surface charge distribution, promoting stronger electrostatic attraction. These synergistic effects significantly improve the composite adsorption capacity for aromatic and charged pollutants (Wu et al. 2015).

**Fig. 6:**
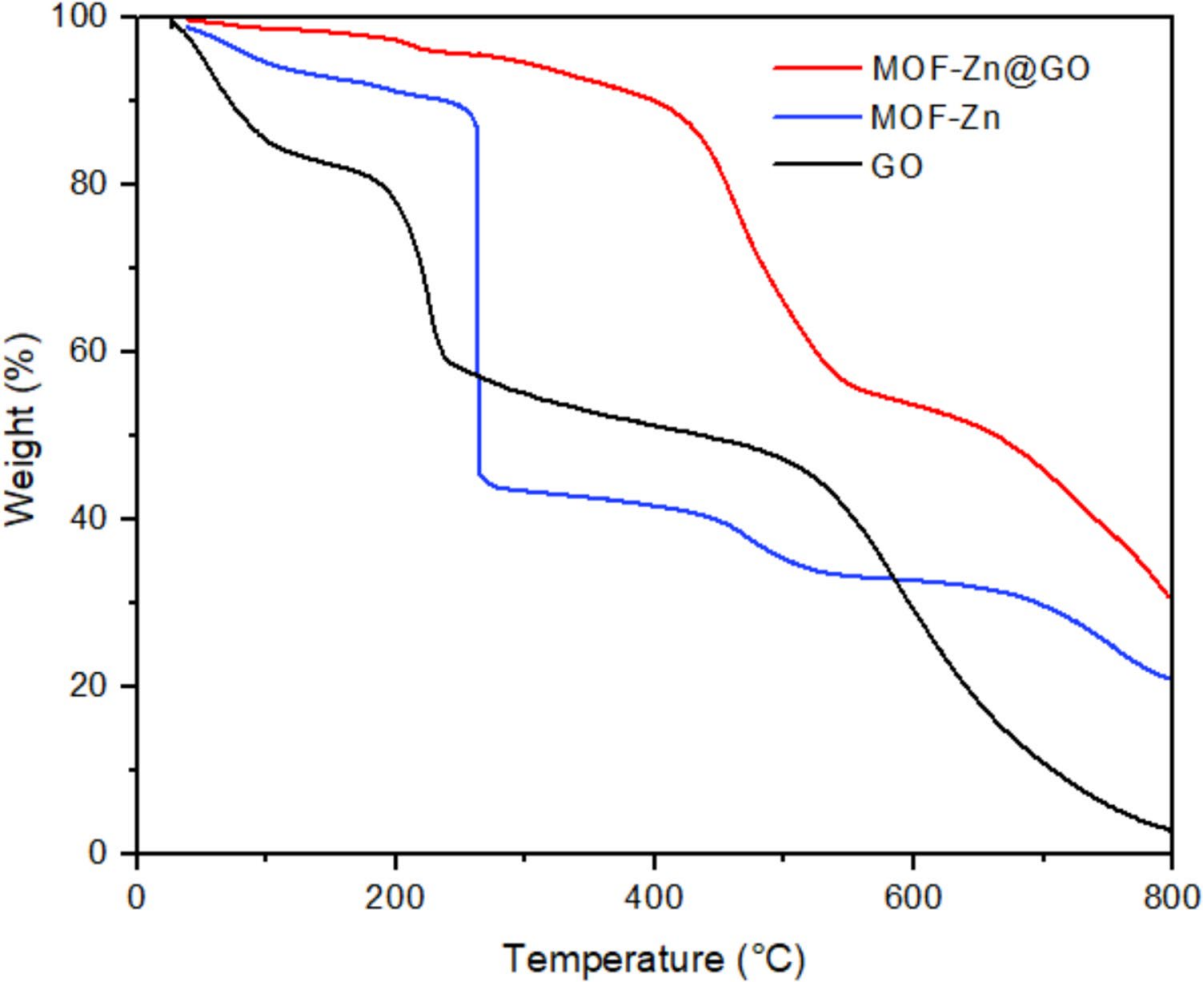
TGA of MOF-Zn, GO, and MOF-Zn@GO

Thermogravimetric analysis (TGA) performed in nitrogen atmosphere up to 800 °C provided insights into the thermal stability and compositional characteristics of MOF-Zn, GO and MOF-Zn@GO materials (Fig. 6). The MOF-Zn shows two major weight loss events: a small initial loss below 150 °C likely due to solvent evaporation, and a sharp decomposition between 250 and 350 °C, corresponding to the breakdown of the organic linker, leaving a residual mass around 40%. GO presents a multistep degradation profile, with significant weight loss between 150 and 250 °C associated with labile oxygenated functional groups, and further degradation up to 600 °C, resulting in a lower final residue. The MOF-Zn@GO composite displays improved thermal stability compared to its components, with a more gradual and extended weight loss spanning from 200 to 700 °C. This broadened decomposition range and delayed onset of major weight loss suggest strong interfacial interactions between MOF-Zn and GO (Shah et al. 2021). The integration of GO into the MOF-Zn structure does not significantly alter the composite’s decomposition profile but instead leads to an extended degradation range and gradual weight loss. This behavior suggests higher thermal durability and better compatibility between the MOF and GO phases, resulting in an improvement in the overall thermal stability of MOF-Zn@GO. This improved thermal robustness underscores the potential applicability of MOF-Zn@GO in environments requiring elevated thermal resistance, where GO functionalization contributes to maintaining the structural integrity and stability of the composite under thermal stress (Nam et al. 2024).

**Fig. 7:**
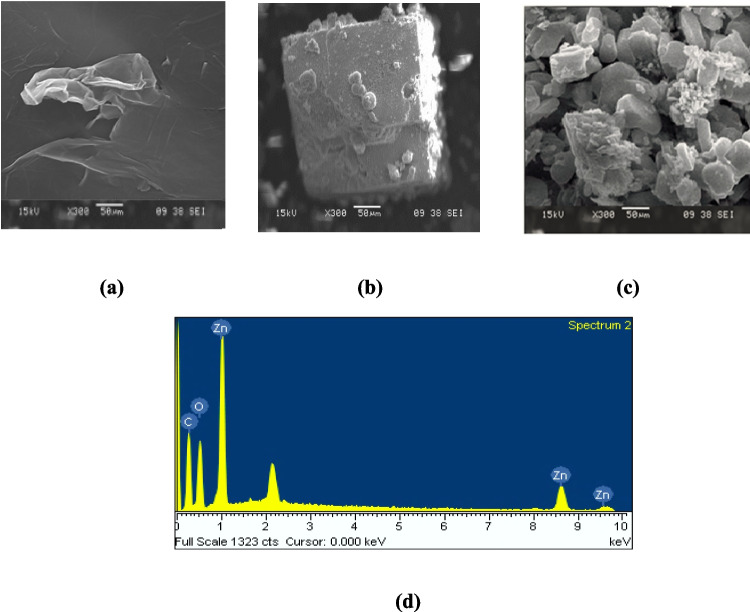
SEM images of **a** GO, **b** MOF-Zn, **c** MOF-Zn@GO, and **d** EDX of MOF-Zn@GO

The morphology of the synthesized GO, MOF-Zn, and MOF-Zn@GO samples was examined using scanning electron microscopy (SEM). GO (Fig. 7a) exhibits its typical wrinkled and layered sheet-like structure, indicative of stacked GO nanosheets with high surface area and abundant oxygen-containing functional groups. In contrast, MOF-Zn (Fig. 7b) displays well-formed tetragonal crystals with smooth surfaces and a uniform particle size distribution ranging from 50 nm, consistent with the expected morphology of Zn-based metal–organic frameworks. Upon incorporation of GO, the morphology of MOF-Zn@GO (Fig. 7b, c) changes significantly. The composite exhibits smaller and irregularly shaped particles, with reduced sizes and a clear loss of the original tetragonal geometry. This transformation can be attributed to the interaction between the functional groups on GO and the Zn metal centers during the synthesis process, which alters the nucleation and crystal growth pathways. The GO sheets likely act as heterogeneous nucleation sites, promoting the formation of smaller MOF domains with rougher surfaces and increased aggregation (Liu et al. 2024a, b). The inclusion of GO not only influences particle shape but also plays a role in structuring the composite framework, resulting in a more densely packed arrangement that may enhance the material's stability and adsorption efficiency (Ahsan et al. 2019).

The EDX analysis of MOF-Zn@GO (Fig. 7d) displays prominent peaks at 8.6 keV and 9.6 keV; these signals confirm the presence of zinc within the composite structure. In addition to Zn, the spectrum also shows characteristic peaks for carbon and oxygen, which are attributed to the organic linkers of the MOF and the graphene oxide sheets. The simultaneous detection of Zn, C, and O elements provides strong evidence of the successful integration of MOF-Zn with GO, indicating effective hybridization and the formation of a stable composite material. The structural integrity and defined morphology of MOF-Zn@GO suggest significant potential for applications in pollutant removal and catalysis, where well-organized, stable structures are crucial for performance (Hoseinzadeh et al. 2021).

#### Surface and textural properties

**Fig. 8:**
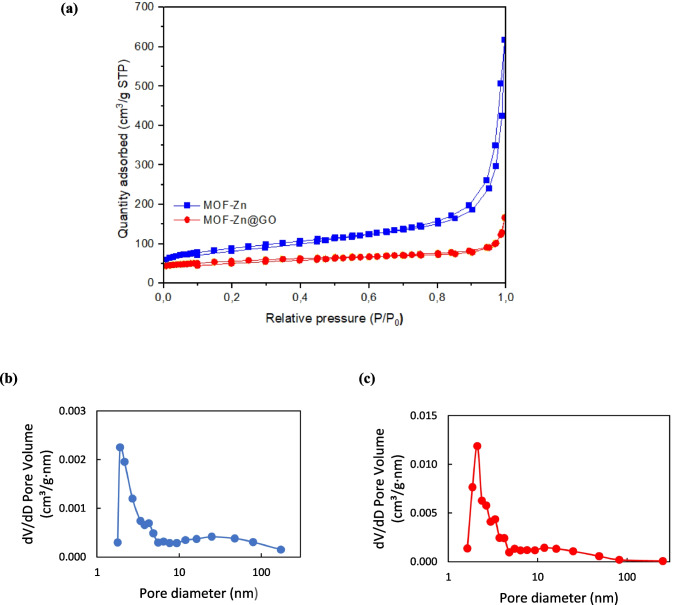
**a** N_2_ adsorption–desorption isotherms of MOF-Zn and MOFs-Zn@GO, **b** pore size distribution pattern of MOF-Zn, **c** pore size distribution pattern of MOF-Zn@GO

The specific surface areas of MOF-Zn and MOF-Zn@GO were analyzed using the Brunauer–Emmett–Teller (BET) method and are shown in Table 2. Both materials exhibited a type IV isotherm indicating the presence of mesoporous structures (Fig. 8a). The surface area of the MOF-Zn@GO composite was 329.7 m^2^ g⁻^1^, with an associated pore volume of 0.45 cm^3^ g⁻^1^. In contrast, the unmodified MOF-Zn displayed a considerably higher surface area of 1100 m^2^ g⁻^1^ and a pore volume of 1.02 cm^3^ g⁻^1^. The average pore diameter was found to be 1.9 nm for MOF-Zn and 2.9 nm for MOF-Zn@GO (Fig. 8b, c). The reduction in surface area and pore volume in the MOF-Zn@GO composite is primarily attributed to the partial occupation of the MOF’s internal pores by functional groups from the ligand and GO. The integration of GO may induce structural imperfections or lead to the aggregation of GO layers, which further contributes to the reduction in surface area by partially blocking or obstructing the MOF’s intrinsic pore framework (Mohd et al. 2022).

**Table 2:** BET and BJH analysis results of the MOF-Zn and MOF-Zn@GO

MOF	Pore volume (cm^3^ g^−1^)	BET surface area (m^2^ g^−1^)	Pore diameter (nm)
MOF-Zn	1.02	1100.0	1.9
MOF-Zn@GO	0.45	329.7	2.1

**Fig. 9:**
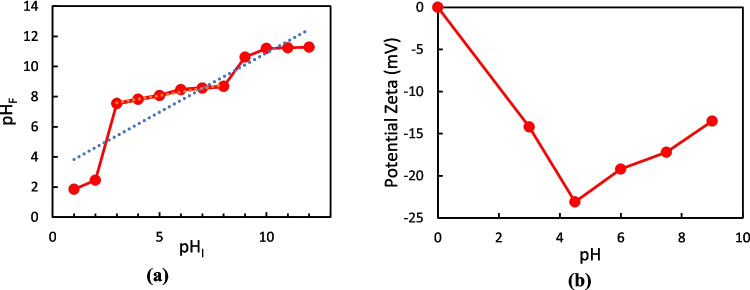
**a** Evaluation of pHpzc and **b** zeta potential of the MOF-Zn@GO

To investigate the surface charge characteristics of the MOF-Zn@GO adsorbent, the pHpzc was determined. Figure 9a presents the plot of initial pH (pHᵢ) versus final pH (pH_f_) after equilibrium. The pHpzc is identified at the intersection point of the pH*ᵢ* = pH_*f*_ line, which corresponds to approximately 6.8. This value indicates that the surface of MOF-Zn@GO is positively charged at pH values below 6.8, favoring the adsorption of anionic species, and at pH values above 6.8, the surface becomes negatively charged, which enhances interactions with cationic pollutants. This information is essential for predicting the adsorption of MOF-Zn@GO behavior under varying pH conditions. The zeta potential measurements of MOF-Zn@GO materials, shown in Fig. 9b, provide critical insights into the charge surface characteristics and their implications for adsorption performance. The MOF-Zn@GO shows a significantly more negative zeta potential of − 23.1 mV. This marked shift in surface charge is attributed to the integration of GO within the MOF structure, particularly due to the presence of oxygen-containing functional groups, namely, carbonyl, epoxy, and hydroxyl groups on the GO surface (Ploychompoo et al. 2021).

The increase in negative surface charge following GO functionalization has several important implications. The MOF-Zn@GO composite possesses sufficient surface charge to remain dispersed, particularly at slightly acidic to neutral pH values. The GO layer, rich in oxygen-containing groups, enhances the electrostatic interactions between MOF-Zn@GO and the contaminants, thereby facilitating improved adsorption. This enhanced interaction is particularly advantageous in aqueous media, where electrostatic forces play a significant role in the adsorption process, and can be modulated by adjusting the solution pH. At higher pH values, for example, the deprotonation of functional groups on both the contaminant molecules and the hybrid MOF surface can lead to stronger electrostatic attraction, optimizing the material’s adsorption capacity (Sun et al. 2020). Although the surface area and pore volume decrease upon GO incorporation, the presence of GO introduces additional functional groups that may enhance the adsorption performance through improved surface interactions. Studies have shown that GO’s layered structure, with its abundant functional groups, can enhance the binding affinity of MOFs toward various organic pollutants, including pharmaceuticals and industrial plasticizers, thus expanding its application potential in water treatment (Ding et al. 2025).

### Adsorption performance

The adsorption performance of MOF-Zn@GO for emerging pollutants (EPs) was systematically evaluated by studying the effect of key parameters such as pH, initial pollutant concentration, sorbent dosage, and eluent volume. The enhanced adsorption capacity is attributed to the synergistic integration of the porous MOF structure with graphene oxide, which introduces additional functional groups (e.g., hydroxyl, carboxyl, and epoxy) that improve interaction with the pollutants.

**Fig. 10:**
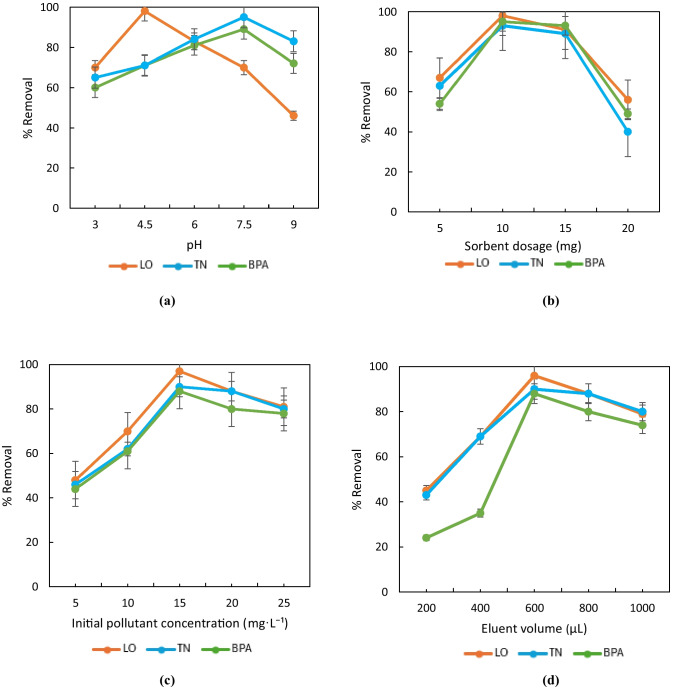
Adsorption performance, effects **a** pH, **b** sorbent dosage, **c** initial pollutant concentration, and **d** eluent volume

The pH of the solution plays a crucial role in determining the surface charge of the adsorbent, the ionization state of the pollutants, and the electrostatic interactions between them. Adsorption experiments were performed across a pH range of 3 to 9. The experiment was performed by adding 5 mg MOF-Zn@GO, EPs solution at different concentrations, and continuous stirring at 25 °C for 3 h. (Fig. 10a). The results indicated that pH significantly influenced the adsorption capacities for all three pollutants. LO showed the highest adsorption efficiency at pH 4.5, while BPA and TN exhibited optimal adsorption at pH 7.5. At pH 4.5, losartan (pKa ≈ 5.5) is predominantly in neutral form but retains polar functional groups, such as carboxylic acids and tetrazole rings, capable of forming strong hydrogen bonds and π–π interactions with the MOF-Zn@GO surface. Although the pHpzc of the MOF-Zn@GO is 6.8, zeta potential measurements reveal a net negative surface charge of − 23.1 mV at this pH, indicating the presence of deprotonated GO groups. This surface chemistry creates a favorable environment for multiple interaction mechanisms, such as hydrogen bonding, π–π stacking, and electrostatic attractions with the losartan molecule. These synergistic interactions significantly enhance the affinity of LO toward the MOF-Zn@GO surface, explaining the maximum adsorption efficiency observed at pH 4.5. In contrast, TN (pKa ≈ 7.9) and BPA (pKa ≈ 9.6), evaluated at pH 7.5, are also largely neutral, but under conditions where the MOF-Zn@GO surface is close to or slightly above its pHpzc.

This results in a near-neutral or slightly negative surface charge, diminishing the role of electrostatic interactions. Consequently, the adsorption of these compounds is primarily governed by nonspecific interactions such as hydrophobic effects and π–π stacking, which are generally weaker and less directional. The comparatively lower adsorption observed for BPA and TN reinforces the importance of electrostatic complementarity and specific functional group interactions in driving the high affinity of losartan under acidic conditions. However, excessively high pH levels may cause the pollutants to compete with hydroxide ions, decreasing adsorption efficiency (Saini et al. 2024).

The effect of MOF-Zn@GO dosage on the removal efficiency of emerging contaminants (EP) was systematically evaluated by varying the sorbent amount from 5 to 20 mg. The experiment was carried out with an initial contaminant concentration of 10 mg L⁻^1^, at pH values of 4.5 and 7.5, under continuous stirring at 25 °C for 3 h (Fig. 10b). Removal efficiency (%) increased significantly up to 10 mg for all analytes, which is attributed to the greater availability of active binding sites on the adsorbent surface. However, a further increase in dosage above 10 mg resulted in a decrease in adsorption capacity (*Qe*, mg g⁻^1^), a phenomenon often associated with particle agglomeration that reduces accessible surface area and clogs internal pores. Furthermore, when contaminant concentration remains constant, excess sorbent material leads to underutilization of the active sites, which reduces the calculated *Qe* due to mass dilution (Kaur et al. 2023; Zhang et al. 2022). The optimal dose was found to be 10 mg for LO, BPA, and TN, achieving removal efficiencies of approximately 97%, 95%, and 92%, respectively. Increasing the dose above this point did not produce a significant improvement in removal, indicating that the system likely reaches equilibrium due to site saturation or diffusion limitations. These findings demonstrate the high efficiency of MOF-Zn@GO at low doses, highlighting its potential for sustainable and cost-effective applications in water treatment.

The experiments were carried out with initial concentrations that ranged between 5 and 25 mg L⁻^1^ (Fig. 10c). The results showed that, as the initial pollutant concentration increased, the amount of adsorbed contaminant per unit mass of MOF-Zn@GO (*Qₑ*) also increased, reflecting increased interaction between contaminants and available active sites. However, the adsorption efficiency (% removal) decreased slightly at higher concentrations, which can be attributed to saturation of the adsorbent active sites. At lower concentrations, the availability of adsorption sites was sufficient to adsorb a significant fraction of the contaminants, resulting in high removal efficiency (Li et al. 2023).

Eluent volume is a crucial parameter, as it determines the effectiveness of contaminant recovery and reuse of the adsorbent material. The elution experiments were carried out using ACN as eluent, with volumes ranging from 200 to 1000 µL (Fig. 10d). The results indicated that increasing the eluent volume improved the desorption efficiency of contaminants from the surface of MOF-Zn@GO. At lower eluent volumes, incomplete desorption was observed, probably due to insufficient contact of the solvent with the adsorbed contaminants. However, the volume of 600 µL was fixed, and maximum desorption efficiency was achieved, allowing for almost complete recovery of pollutants. Further increases in eluent volume did not result in significant improvements in desorption, suggesting that saturation of eluent capacity had been reached. Furthermore, excessive eluent volumes could dilute the recovered contaminants, complicating downstream analysis and reuse of the adsorbent material (Mahmad et al. 2022).

The adsorption kinetics of EPs on MOF-Zn@GO were analyzed to determine the adsorption rate and mechanism. The kinetic study provides insights into how rapidly the pollutants interact with the adsorbent surface and reach equilibrium. The experimental data and equations for kinetic models are found in Table 3.

**Table 3:** Parameters of kinetic models

Pseudo–first order ln (*q*_*e*_* − q*_*t*_) = ln*q*_*e*_* − k*_*1*_*t*				
	***K***_**1**_** (min**^**−1**^**)**	***R***^**2**^		**AICc**
**LO**	0.033	0.897		15.8
**BPA**	0.012	0.831		17.9
**TN**	0.037	0.888		16.7
**Pseudo–second order**$\frac{t}{{q}_{t}}= \frac{1}{{K}_{2}{q}_{e}^{2}}+ \frac{t}{{q}_{e}}$				
	***K***_**2**_** (mg g**^**−1**^** min**^**−1**^**)**	***R***^**2**^		**AICc**
**LO**	1.98 × 10^−4^	0.995		7.67
**BPA**	1.42 × 10^−4^	0.991		9.41
**TN**	3.22 × 10^−4^	0.995		8.89
**Elovich model *q***_***t***_** = **$\frac{1}{\beta }\text{ln}\left(\alpha \beta \right)+ \frac{1}{\beta }\text{ln}t$				
	***K***_***E***_** (mg g**^−**1**^** min**^**−1**^**)**	**β (g mg**^**−1**^**)**	***R***^**2**^	**AICc**
**LO**	3.645	0.812	0.912	33.3
**BPA**	2.636	0.603	0.901	38.5
**TN**	3.273	0.801	0.916	36.5
**Intra-particle diffusion *q***_***t***_** = *k***_***id***_***t***^**1/2**^** + *C***				
	***Kₚ* (mg g**^**−1**^** min**^**−1**^**ᐟ**^**2**^**)**	***R***^**2**^		**AICc**
**LO**	11.19	0.792		24.0
**BPA**	17.89	0.585		32.3
**TN**	5.72	0.789		27.4

The adsorption kinetics of the EPs were best described by the pseudo-second-order model, with excellent correlation coefficients (*R*^2^ = 0.991–0.995), indicating that chemisorption dominates the process through electron exchange or sharing between MOF-Zn@GO and the adsorbates (Zhao et al. 2022). Among the contaminants, TN exhibited the highest rate constant (*k*₂), followed by LO and BPA, due to its smaller size and higher polarity facilitating faster diffusion and adsorption (De Oliveira et al. 2024). Although LO adsorbs more slowly, its greater capacity arises from stronger π–π interactions, hydrogen bonding, and van der Waals forces with MOF-Zn@GO. TN adsorption is predominantly surface-controlled with rapid chemisorption, while LO involves combined diffusion- and surface-controlled mechanisms. The Elovich model fit indicates heterogeneous adsorption sites consistent with GO-induced structural complexity.

The adsorption isotherms of LO, BPA, and TN on MOF-Zn@GO were investigated to elucidate the adsorption mechanisms and to quantify the capacity of the material for each contaminant. The experiments were performed by varying the initial concentrations of the contaminants (50–250 mg L⁻^1^) while keeping the temperature, pH, and contact time constant. Table 4 shows the data were fitted to both the Langmuir, the Freundlich, and the Temkin isotherm models. The results showed that the Freundlich model provided a better fit for the experimental data, as indicated by the higher correlation coefficients (*R*^2^) compared to the Langmuir model. This suggests that the adsorption process on MOF-Zn@GO occurs on a heterogeneous surface with variable energy sites and involves multilayer adsorption. Freundlich constants, including adsorption capacity (*K*_*F*_) and adsorption intensity (*n*), further confirmed the strong affinity of the material for LO, BPA, and TN. The adsorption capacity values obtained from the Freundlich model were 395 mg g⁻^1^ for LO, 275 mg g⁻^1^ for BPA, and 300 mg g⁻^1^ for TN.

**Table 4:** Isotherm modeling results for the adsorption of pollutants onto MOF-Zn@GO

**Freundlich** Log *qe* = log${K}_{f}$ + $\frac{1}{n}$log*Ce*				
**1/*n***_***F***_		***K***_***F***_**/(L mg**^**−1**^**)**	***R***^**2**^	***Q***_**max**_**(mg g**^**−1**^**)**
**LO**	0.60	0.16	0.997	395.1
**BPA**	0.40	0.11	0.993	275.2
**TN**	0.58	0.16	0.997	300.3
**Langmuir **$\frac{{C}_{e}}{{q}_{e}}$** = **$\frac{1}{b\cdot q\text{max}}$** + **$\frac{{C}_{e}}{q\text{max}}$				
***q***_**max/**_**(mg g**^**−1**^**)**		***K***_***L***_**/(L mg)**^**−1**^	***R***^**2**^	
**LO**	− 7.343	− 1.6 × 10^**−**6^	0.972	
**BPA**	− 3.986	− 6.3 × 10^**−**6^	0.948	
**TN**	− 7.368	− 1.4 × 10^**−**6^	0.973	
**Temkin *qe* = *B* ln(*AT*) + *B* ln*C***				
	***B***_***T***_** (j mol**^**−1**^**)**	***K***_***T***_** (L mg**^**−1**^**)**	***R***^**2**^	
**LO**	5.247	0.77	0.936	
**BPA**	4.748	0.87	0.897	
**TN**	4.855	0.79	0.904	

The thermodynamic parameters for the adsorption of emerging pollutants (EPs) LO, BPA, and TN on MOF-Zn@GO were evaluated to gain insights into the nature of the adsorption process. Based on the van’t Hoff equation (Eqs. 5 and 6), key thermodynamic parameters, enthalpy change (*ΔH*), entropy change (*ΔS*), and Gibbs free energy change (*ΔG*) were calculated from adsorption experiments conducted at different temperatures (298 K, 308 K, and 318 K) as shown in Table 5.

5 $$\text{ln}\frac{{q}_{e}}{{C}_{e}}=\frac{\Delta S}{R}-\frac{\Delta H}{RT}$$

6 $$\Delta G=\Delta H-T\Delta S$$

where *R* is a universal gas constant (8.314 J/mol K) and *T* is a temperature (K).

**Table 5:** Thermodynamic parameters for the adsorption of pollutants on MOF-Zn@GO

EPs	Temperature (K)	*ΔG*°	*ΔH*° (kJ mol^−1^)	*ΔS*° (J mol^−1^ K^−1^)
**LO**	298	− 21.65	65.36	102.32
	308	− 21.34		
	318	− 21.86		
**BPA**	298	− 20.32	63.87	118.37
	308	− 20.56		
	318	− 21.13		
**TN**	298	− 21.35	64.54	103.43
	308	− 21.78		
	318	− 22.21		

The negative *ΔG* values at all temperatures indicated that the adsorption of EP onto MOF-Zn@GO is a spontaneous process. As the temperature increased, the magnitude of *ΔG* decreased slightly, suggesting a higher thermodynamic favorability of adsorption at higher temperatures, possibly due to the increase in molecular motion and interaction energy. This balance reflects competing enthalpic and entropic effects influencing the adsorption mechanism.

The positive *ΔH* values obtained for the adsorption of LO, BPA, and TN onto MOF-Zn@GO indicate an endothermic process, with adsorption capacity increasing at higher temperatures. Thermal energy enhances pollutant mobility and diffusion, facilitating penetration into pores and interaction with active sites. Elevated temperatures also help overcome activation energy barriers, promoting stronger adsorbate-adsorbent interactions such as coordination bonds and π–π stacking. This temperature-dependent behavior benefits water treatment under variable thermal conditions by improving adsorption kinetics and equilibrium capacity. However, material stability and operational costs impose practical limits, requiring an optimal temperature range to ensure performance and durability in real applications. Positive *ΔS* values for all three contaminants highlight increased randomness at the solid–liquid interface during adsorption, likely due to displacement of water molecules by contaminants and structural rearrangements within the MOF-Zn@GO framework (Zhang et al. 2022).

LO, BPA, and TN were selected as representative EPs in this study due to their environmental relevance and distinctive physicochemical characteristics. LO, a pharmaceutical compound with a relatively high molecular weight, contains multiple aromatic rings, a tetrazole group (acidic), and a potassium counterion that enhances its aqueous solubility and ionic character, making it representative of drugs with both hydrophilic and hydrophobic functionalities. BPA is a phenolic compound comprising two hydroxylated aromatic rings linked by an isopropylidene bridge; its moderate hydrophobicity and capacity to form hydrogen bonds make it a model endocrine disruptor with high adsorption potential via π–π interactions and hydrogen bonding. TN, a chlorinated diphenyl ether, features hydrophobic aromatic rings, electron-withdrawing chlorine substituents, and a phenolic group, contributing to its pKa-dependent solubility and reactivity. The chemical diversity of these compounds enables a comprehensive evaluation of the MOF-Zn@GO adsorption performance under various interaction mechanisms, supporting its potential for the removal of structurally and functionally diverse organic pollutants.

**Fig. 11:**
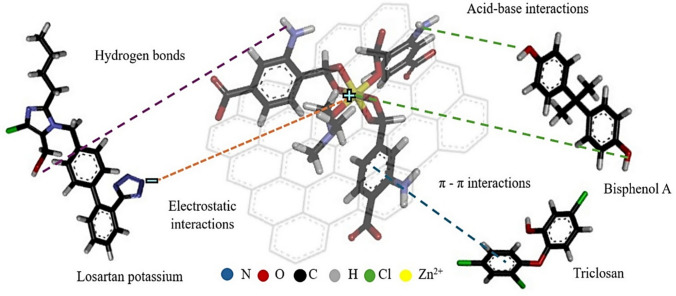
Proposed adsorption mechanism of LO, BPA, and TN on MOF-Zn@GO

The adsorption mechanism of LO, BPA, and TN onto MOF-Zn@GO involves a combination of physicochemical interactions that enable the efficient removal of these emerging pollutants from aqueous solutions (Fig. 11). The structure and functionalization of MOF-Zn@GO play a crucial role in facilitating these interactions. The FTIR spectrum of MOF-Zn@GO confirms the presence of key functional groups such as hydroxyl (–OH), carboxyl (–COOH), and aromatic C = C bonds, originating from both the GO sheets and the MOF structure. These functionalities are directly involved in the adsorption mechanism by enabling specific chemical interactions with pollutant molecules. The hydroxyl and carboxyl groups can form directional hydrogen bonds or act as electron donors in coordination with unsaturated Zn^2^⁺ sites within the framework. Simultaneously, the conjugated π systems present in both the MOF linkers and GO facilitate π–π stacking interactions with aromatic contaminants.

XPS analysis confirms the presence of Zn^2^⁺ centers (Zn 2p), oxygenated groups (C1s, O1s), and nitrogen-containing organic linkers (N1s), which can contribute to electron-donor–acceptor interactions. These surface functionalities enable coordination bonding with electron-rich pollutants, hydrogen bonding, and π–π interactions, supporting a chemisorption mechanism at the MOF-Zn@GO interface. This interaction is particularly important for LO and BPA, which contain functional groups capable of forming strong hydrogen bonds with the active sites on the composite. The aromatic rings present in LO, BPA, and TN interact with the conjugated π-electron system of graphene oxide through π-π stacking. This interaction is enhanced by the large surface area of graphene oxide, which provides multiple adsorption sites for aromatic pollutants (Mehdi and Aravamudan 2024).

**Fig. 12:**
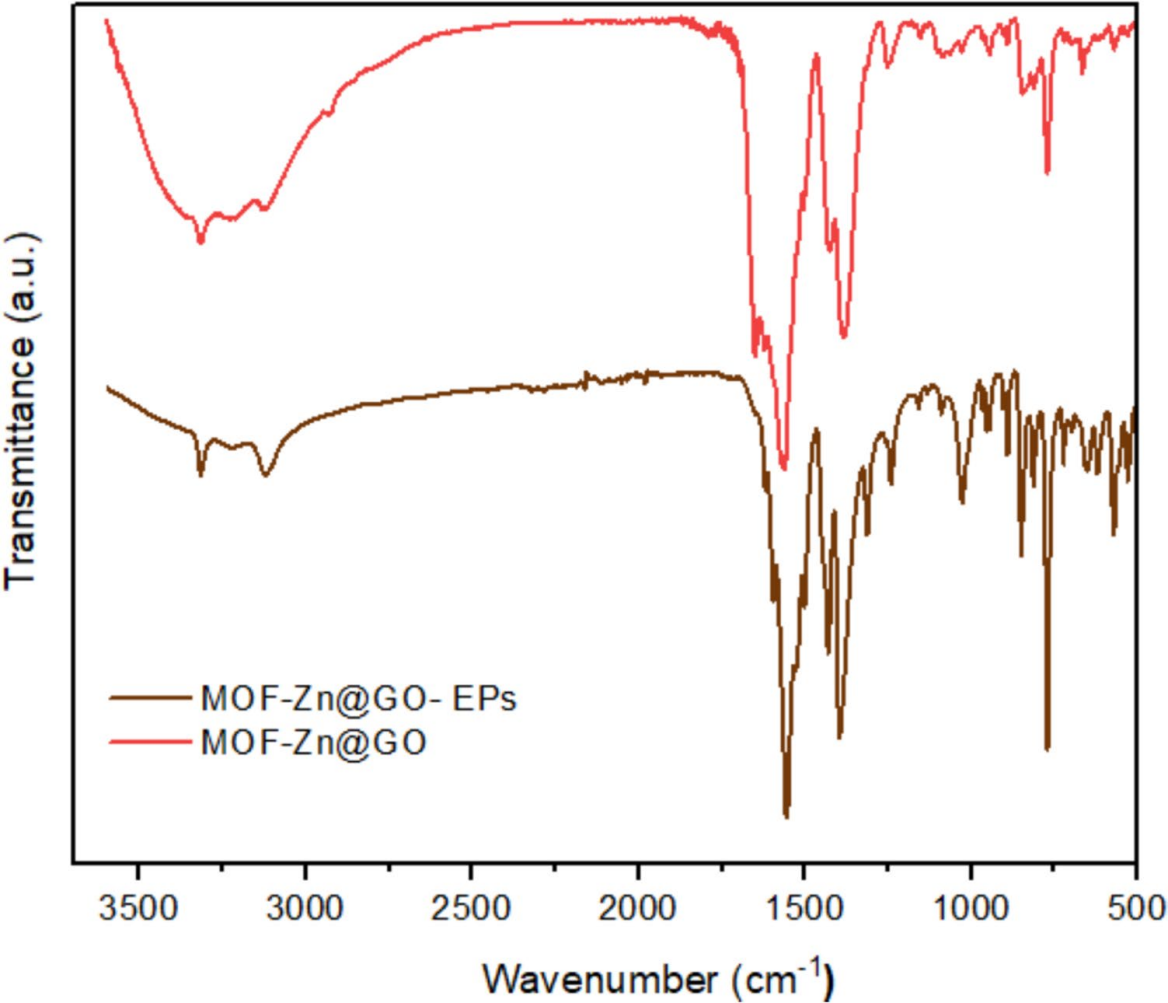
FTIR spectra of MOF-Zn@GO before and after adsorption (MOF-Zn@GO-EPs) of emerging contaminants (LO, BPA, and TN)

The FTIR spectra of MOF-Zn@GO before and after adsorption of emerging contaminants (LO, BPA, and TN), shown in Fig. 12, provide important information about the structural and chemical stability of the material. The characteristic bands associated with the functional groups of MOF-Zn@GO remain evident after the adsorption process, indicating that the structure retains its integrity. Notably, the broad band around 3300 cm⁻^1^, attributed to O–H stretching vibrations, becomes more prominent in the post-adsorption spectrum, suggesting increased hydrogen bonding interactions due to the presence of adsorbed molecules. The peak near 1600 cm⁻^1^, corresponding to C = O or C = C vibrations of carboxylate groups or aromatic bonds, also shifts slightly and changes in intensity, likely due to π–π interactions and electrostatic attractions between the contaminants and the MOF-Zn@GO surface. In the fingerprint region (1200–600 cm⁻^1^), the preservation of the bands associated with Zn–O vibrations confirms that the inorganic nodes of the MOF are not altered by the adsorption process. While small shifts and intensity changes are observed in some regions, key functional groups do not disappear, indicating that the material remains chemically stable, highlighting that the Zn@GO-MOF maintains its structural and functional integrity upon adsorption, with spectral modifications due to interactions with contaminants. These findings support the material’s potential for reuse in water treatment applications.

In water treatment, the presence of inorganic components, such as alkali and alkaline earth metal ions, or common anions, can influence adsorption behavior through competitive interactions or changes in surface charge distribution. However, the structural features of MOF-Zn@GO, which combine the high surface area and porosity of Zn-based metal–organic frameworks with the π-conjugated functionalized domains of GO, favor stable adsorption performance in complex aqueous matrices. The endothermic nature of the process can help maintain or even enhance pollutant sorption under thermally variable conditions, such as those present in wastewater effluents or thermally impacted natural waters, highlighting the potential applicability of MOF-Zn@GO in real sample purification systems (Abbasi et al. 2023).

**Fig. 13:**
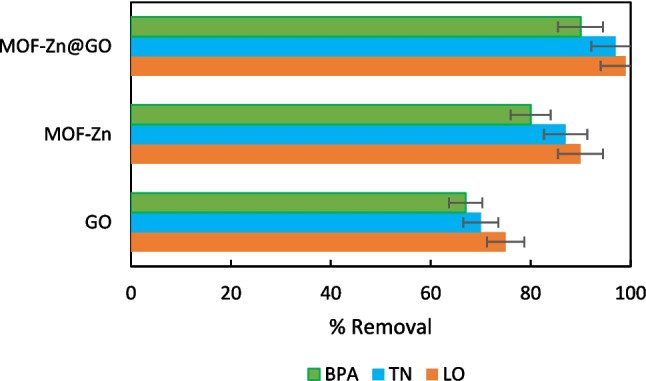
Adsorption efficiency of MOF-Zn@GO, MOF-Zn, and GO for the removal of LO, BPA, and TN

The adsorption of pollutants is influenced by the surface charge of MOF-Zn@GO, which is pH-dependent. At the pH levels studied, the surface of the adsorbent exhibits a negative charge due to the deprotonation of functional groups, promoting electrostatic attraction with positively charged species like LO. The unsaturated Zn(II) sites in the MOF framework provide additional active sites for adsorption, particularly through coordination bonds with functional groups on the pollutants, such as hydroxyl or carboxyl groups. Physical interactions such as van der Waals forces also contribute to the adsorption process, particularly in the case of BPA, where physisorption plays a significant role (Yu et al. 2022). The adsorption efficiency of MOF-Zn@GO, MOF-Zn, and GO for the removal of LO, BPA, and TN was evaluated under the same set of experimental conditions (Fig. 13).

The superior adsorption performance of MOF-Zn@GO compared to MOF-Zn and GO individually is due to the synergistic integration of the MOF’s high porosity and functional groups of GO. The hybrid material benefits from enhanced surface area, better dispersion of active sites, and strong pollutant-specific interactions such as hydrogen bonds, π–π stacking, and electrostatic forces (Zheng et al. 2022). This demonstrates that MOF-Zn@GO is a highly effective adsorbent for removing emerging pollutants from aqueous solutions. The enhanced selectivity and structural stability imparted by GO improve the overall performance of the adsorbent in practical applications, potentially reducing the total amount of material required to achieve target contaminant removal. The higher adsorption efficiency of MOF-Zn@GO suggests fewer cycles and less frequent replacement, which can lower operational costs and waste generation over time. Therefore, MOF-Zn@GO presents advantages in sustainability and cost-effectiveness for water purification applications.

A comparative analysis with other reported MOF-based adsorbents for emerging pollutant removal is provided in Table 6. This comparison highlights the adsorption capacities, target pollutants, and key mechanisms involved, emphasizing the advantages of the present work in terms of high efficiency and selectivity.

**Table 6:** Comparison of MOF-based adsorbents for the removal of emerging pollutants

MOF	Target pollutant(s)	Max. adsorption capacity (mg g⁻^1^)	pH	Adsorption mechanism	Ref
Fe₃O₄@MIL-53(Al)	BPA	160.9	4.5	π–π interactions, H-bonding	Zhang et al. 2021a, b
ZIF-8	Tetracycline	359.61	6.0	Electrostatic and π–π stacking	Li et al. 2024
MIL-101(Cr)	Bisphenol A	252.5	6.0	H-bonding, pore filling	Qin et al. 2014
Alg@MOF-rGO	Tetracycline (TC) Ciprofloxacin (CIP)	43.76 40.76	7.0	Electrostatic interaction, pore filling, H-bonding, and π–π stacking	Kim et al. 2022
ZIF-8@GO	Methylene blue (MB), methyl orange (MO)	87.39 (MB), 82.78 (MO)	6–7	Electrostatic interactions, π–π stacking	Younis et al. 2025
MOF-Zn@GO	LO, BPA, TN	395 (LO) 275 (BPA) 300 (TN)	4.5 7.5	H-bonding, π–π interactions, GO synergy	This study

The extraction method was applied, and the adsorption method demonstrated excellent linearity within the concentration range of 0.1 to 50 µg mL⁻^1^ of EPs. The correlation coefficients (*r*^2^) exceeded 0.998, reflecting strong consistency between the pollutant concentration and the analytical response. The regression equation for the process was determined, where *y* represents the analytical signal and *x* is the pollutant concentration. The quantitative analytical parameters for the adsorption of LO, BPA, and TN on MOF-Zn@GO were evaluated and are summarized in Table 7.

**Table 7:** Validation parameters

Analytes	Regression equation	*r*^2^	LOD ng mL^−1^	LOQ ng mL^−1^	RSD (*n* = 5) (%)	Recovery (%)
LO	*y* = 15.324*x* − 0.417	0.998	25	80	1.17	97.9
BPA	*y* = 16.152*x* − 6.2552	0.995	50	100	1.85	89.2
TN	*y* = 16.192*x* − 7.8484	0.995	50	100	1.62	90.2

The method demonstrated excellent precision, with a relative standard deviation (RSD) of 0.75% for a pollutant concentration of 1 µg mL⁻^1^ (*n* = 5). This low RSD value indicates the reproducibility and reliability of the adsorption measurements.

**Fig. 14:**
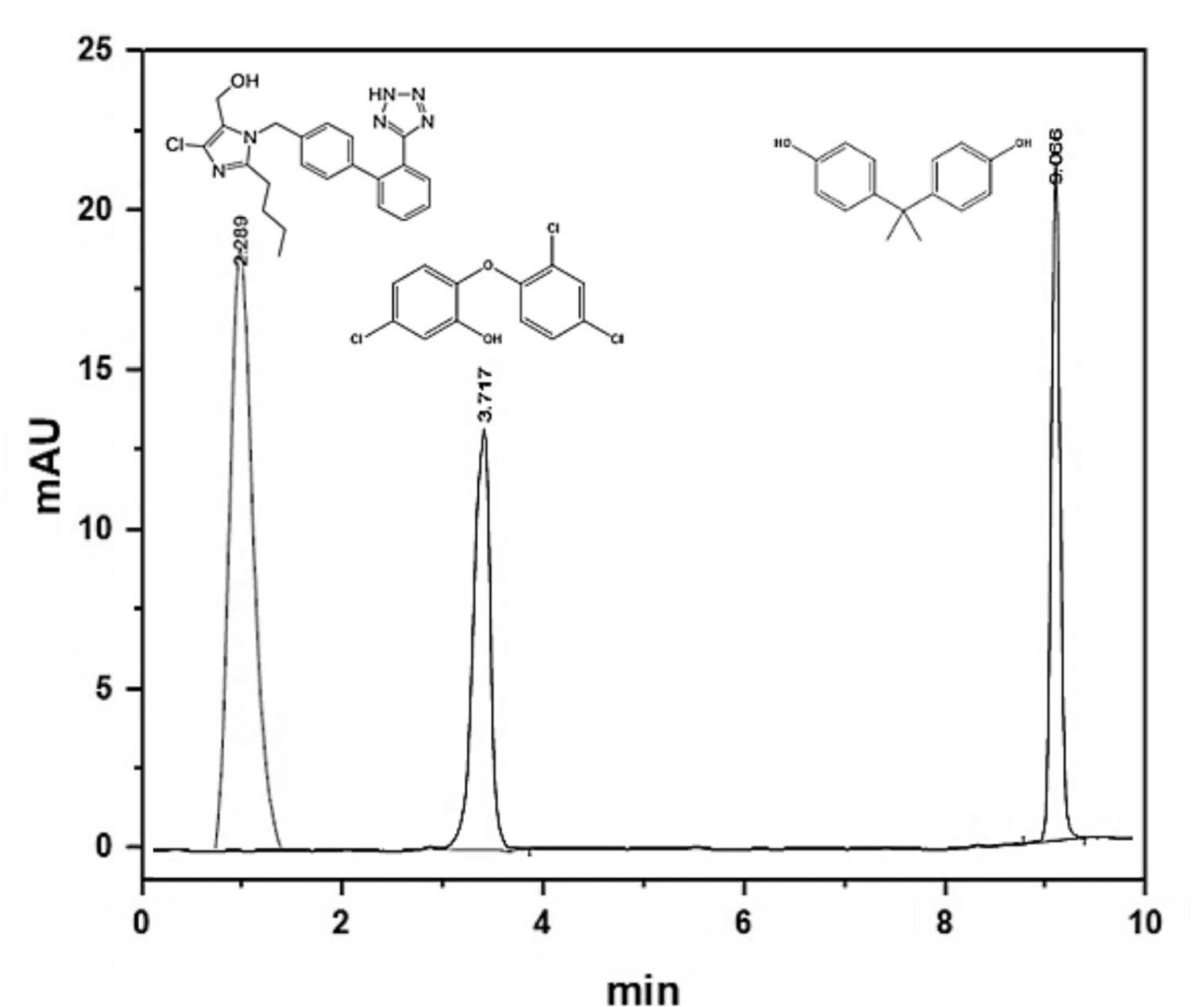
Chromatogram of LO, BPA, and TN

The adsorption performance of MOF-Zn@GO was evaluated in real tap water samples fortified with 15 µg mL⁻^1^ of LO, BPA, and TN. The results demonstrated that MOF-Zn@GO maintained over 95% of its adsorption efficiency for the target pollutants. The presence of competing ions and organic matter in tap water slightly reduced the adsorption capacities compared to tests performed in ultrapure water. This is likely due to the competitive adsorption of natural organic matter (NOM) or other trace contaminants and the influence of dissolved salts on the electrostatic interactions between the MOF-Zn@GO and the contaminants (Wang et al. 2021). Tap water pH (approximately 7.2) was favorable for the adsorption process, as it maintained the ionized states of LO, BPA, and TN, enabling strong interactions with the adsorbent. This result underscores the material's robustness and practical applicability in complex environmental conditions. The chromatogram of tap water samples (Fig. 14) shows LO, BPA, and TN signals of the spiked samples identified by the retention time.

**Fig. 15:**
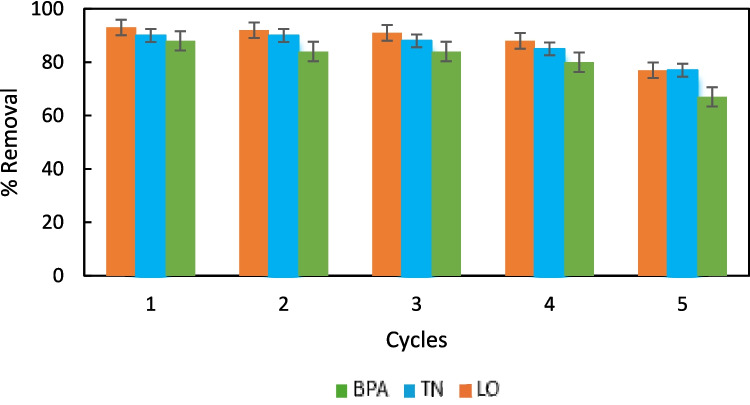
Recyclability for MOF-Zn@GO

The selectivity of MOF-Zn@GO was systematically evaluated through competitive adsorption experiments involving coexisting contaminants such as phenol, benzoic acid, caffeine, and acetaminophen. For the study, 15 µg mL⁻^1^ of these compounds were introduced into solutions containing each emerging contaminant (EP) under the established optimal experimental conditions. The results demonstrated that MOF-Zn@GO maintained its high removal efficiency for LO 98.3%, BPA 88.3%, and TN 96.7% despite the presence of these additional organic contaminants. These results confirm the potential of MOF-Zn@GO for applications in water treatment systems, particularly in scenarios where a wide range of organic contaminants coexist. These quantitative parameters validate the efficiency of MOF-Zn@GO as an adsorbent for EPs, providing a sensitive, precise, and eco-friendly approach for water purification and environmental monitoring applications. Reusability tests were conducted over five consecutive cycles (Fig. 15). After each adsorption process, the spent MOF-Zn@GO was regenerated by desorbing the pollutants using 1 mL of ACN, followed by washing with deionized water to remove residual ACN and pollutants. The regenerated material was then reused under the same adsorption conditions. The results showed that MOF-Zn@GO retained over 90% of its initial adsorption capacity after the first three cycles, demonstrating excellent stability and efficiency. By the fifth cycle, the adsorption capacity decreased slightly, retaining approximately 85% of its original capacity. The slight reduction can be attributed to the gradual loss of active sites or partial degradation of functional groups during repeated cycles (Prasetya and Wöll 2023).

The high recyclability of MOF-Zn@GO reduces the overall cost of water treatment processes by minimizing the need for fresh adsorbent material. Moreover, its effective regeneration with a minimal amount of solvent contributes to environmentally sustainable pollutant removal practices (Zhang et al. 2021a, b). These attributes position MOF-Zn@GO as a promising candidate for scalable and cost-effective environmental remediation, aligning with principles of green chemistry and circular economy in wastewater treatment applications.

## Conclusion

Zn (II)-based metal–organic framework functionalized with GO (MOF-Zn@GO) was synthesized and used as a sorbent MOF-Zn@GO to remove LO, BPA, and TN in aqueous solutions. The synergistic combination of the large surface area of the metal–organic framework and the functional groups of GO enhanced the adsorption process through multiple mechanisms, including hydrogen bonds, π–π stacking, electrostatic interactions, and metal coordination. The adsorption behavior aligns with the Freundlich isotherm model, indicating multilayer adsorption on a heterogeneous surface, and follows pseudo–second order kinetics, highlighting the dominance of chemisorption with a maximum capacity of 395 mg g⁻^1^ for LO, 275 mg g⁻^1^ for BPA, and 300 mg g⁻^1^ for TN. Compared to the individual components (MOF-Zn and GO), the hybrid material exhibits significantly improved performance, confirming the benefits of functionalization. MOF-Zn@GO offers a sustainable and efficient solution for the removal of emerging contaminants, addressing critical challenges in water purification and environmental remediation, demonstrated by the high removal percentages of LO 99.3%, BPA 90.1%, and TN 97.2%. The material’s ability to retain over 85% of its adsorption capacity after five regeneration cycles demonstrates excellent stability and a cost-effectiveness ratio. Its robustness, reusability, and high adsorption efficiency position this material as a viable option for large-scale implementation in advanced water treatment technologies.

Although the MOF-Zn@GO composite demonstrated promising adsorption performance, some limitations were identified, likely due to partial blockage or degradation of active sites. Additionally, adsorption kinetics assessed under controlled conditions may not accurately represent real water systems, where complex matrices with competing ions and variable electrochemical environments prevail. Thus, validation using real-world water samples from relevant sources is essential. To overcome these challenges, future efforts should aim to enhance the structural stability of the composite, potentially through post-synthetic modifications, to preserve its integrity and efficiency across multiple cycles. Improved reusability would reduce material consumption, operational costs, and solid waste generation. Moreover, the potential for regeneration under mild, eco-friendly conditions reinforces the material’s compatibility with green chemistry principles and its suitability for sustainable water treatment applications.
